# Anti-Toxine for Diphtheria

**Published:** 1897-08

**Authors:** 


### ANTI-TOXINE FOR DIPHTHERIA.

"One of the strongest arguments against the anti-toxine treatment of diphtheria is the attitude of those who are living in the hospitals and watching its results, who formerly were earnest advocates of the treatment, and who after experience have from conviction become opposed to it. Dr. William M. Welch (Municipal Hospital of Philadelphia), who has injected over 300 patients, stated that he would not have anti-toxine used on himself if he had diphtheria, that if his children had diphtheria of any type *he would not allow them to be injected*, and if left to act independently and from his own conviction *he would never inject another patient*."

"Dr. Warmuth, Dr. Tyler, and Dr. Bemis (of the Municipal Hospital, Philadelphia), were believers in anti-toxine, and are now opposed to it. Dr. Hardin, of Washington, and Dr. Levy, of Richmond, came to the Willard Parker Hospital thorough believers in anti-toxine, and are now opposed to it. Dr. Warmuth had diphtheria and refused to have anti-toxine used on himself, likewise Dr. Levy and Dr. Hess, of the Willard Parker Hospital, refused to have anti-toxine used.

"Not one of the nurses on the staff of these hospitals would have anti-toxine used on themselves for immunizing purposes. Variot states that the doctors and nurses of the Trouseau Hospital, Paris, would not have anti-toxine used for immunizing purposes, although one of the doctors had severe diphtheria."—*Our Dumb Animals.*

